# Crystal structure of the red light-activated channelrhodopsin Chrimson

**DOI:** 10.1038/s41467-018-06421-9

**Published:** 2018-09-26

**Authors:** Kazumasa Oda, Johannes Vierock, Satomi Oishi, Silvia Rodriguez-Rozada, Reiya Taniguchi, Keitaro Yamashita, J. Simon Wiegert, Tomohiro Nishizawa, Peter Hegemann, Osamu Nureki

**Affiliations:** 1 Department of Biological Sciences Graduate School of Science, The University of Tokyo, Tokyo, 113-0034 Japan; 2 Institute of Biology, Experimental Biophysics, Humboldt-Universität zu Berlin, 10115 Berlin, Germany; 3 Research Group Synaptic Wiring and Information Processing, Center for Molecular Neurobiology Hamburg (ZMNH), 20251 Hamburg, Germany; 4 RIKEN SPring-8 Center, Sayo, 679-5198 Japan; 5 Precursory Research for Embryonic Science and Technology (PRESTO), Japan Science and Technology Agency, Kawaguchi, 332-0012 Japan

## Abstract

Channelrhodopsins are light-activated ion channels that mediate cation permeation across cell membranes upon light absorption. Red-light-activated channelrhodopsins are of particular interest, because red light penetrates deeper into biological tissues and also enables dual-color experiments in combination with blue-light-activated optogenetic tools. Here we report the crystal structure of the most red-shifted channelrhodopsin from the algae *Chlamydomonas noctigama*, Chrimson, at 2.6 Å resolution. Chrimson resembles prokaryotic proton pumps in the retinal binding pocket, while sharing similarity with other channelrhodopsins in the ion-conducting pore. Concomitant mutation analysis identified the structural features that are responsible for Chrimson’s red light sensitivity; namely, the protonation of the counterion for the retinal Schiff base, and the polar residue distribution and rigidity of the retinal binding pocket. Based on these mechanistic insights, we engineered ChrimsonSA, a mutant with a maximum activation wavelength red-shifted beyond 605 nm and accelerated closing kinetics.

## Abstract (web summary)

Channelrhodopsins are light-activated ion channels that mediate cation permeation across cell membranes upon light absorption. Here, the authors report the crystal structure of the most red-shifted channelrhodopsin from the algae *Chlamydomonas noctigama* at 2.6 Å resolution.

## Introduction

The Rhodopsin family is the major photoreceptor family and is widely distributed throughout many lineages of life. Rhodopsins mediate passive or active ion transport or metabotropic reactions in response to light and share a common architecture, consisting of a single polypeptide with 7-transmembrane (7TM) segments (opsin) and a retinal chromophore covalently attached to a conserved lysine residue via a Schiff base. In prokaryotic pumps, light absorption induces retinal isomerization and subsequent transitions through optically distinct intermediates that drive proton, chloride or sodium ion translocation from the intracellular to the extracellular side or vice versa^1,2^, while a similar process induces ion pore formation and passive ion translocation in channelrhodopsins (ChRs)^3,4^. ChRs mediate phototaxis in green algae and are exceptionally popular in neurosciences, where they are used to manipulate neuronal activity with light to study multiple aspects of brain function ranging from synaptic plasticity to network analysis^5,6^. The first structural insights into ChRs were provided by the crystal structure of a ChR chimera, C1C2 (TM1–5 from *Chlamydomonas reinhardtii* ChR1 (*Cr*ChR1) and TM6–7 from *Cr*ChR2)^7^, which revealed a dimeric protein architecture, with the putative ion permeation pathway located in each protomer. The C1C2 structure has enabled the molecular interpretation of a large volume of electrical and spectroscopic data and allowed the rational design of ChR variants with blue-shifted absorption^8^ and charge-inverted anion selectivity^9–12^. Most recently, the structure of *Cr*ChR2 was also reported^13^ and provided further structural information about the ChR family.

While *Cr*ChR2 is still the most commonly employed ChR for cell depolarization, recent sequence mining provided new ChR variants from different algae, with accelerated photocurrent kinetics, different ion selectivity, improved single channel conductance, and shifted peak absorption^14–16^. Given that the low transmission of visible light in organic tissue is one of the major obstacles in optogenetics, red-shifted ChR variants are particularly important, as longer wavelength light allows deeper tissue penetration. *Volvox carteri* ChR1 (*V*ChR1), with peak absorption at 530 nm, was first identified^17^, and several engineered *V*ChR1 constructs, such as C1V1 and ReaChR, were further developed^18–20^. More recently, a ChR from *Chlamydomonas noctigama* (Chrimson) was identified as the variant with the most red-shifted absorption peak at 590 nm^16^, which is more than 40 nm further red-shifted than all other ChRs. Chrimson allowed efficient red-light excitation beyond the operational range of native photoreceptors in adult flies and also enabled independent dual-color experiments in combination with blue-light-activated ChRs. Therefore, Chrimson represents one of the most promising ChRs for both basic neuroscientific research and medical applications. Meanwhile, the remaining blue-light sensitivity of Chrimson demands careful adjustment of light intensities to achieve purposeful combination with blue-light-activated tools, but the low sequence similarity to the structurally-resolved ChRs, such as C1C2 and *Cr*ChR2, has hampered further molecular engineering of Chrimson.

Here we present the crystal structure of Chrimson at 2.6 Å resolution, which reveals (i) the presence of an outer gate occluding the retinal Schiff base from the extracellular solvent, (ii) unique protonation state of the counterion residue destabilizing the protonated retinal Schiff base in the ground state, (iii) highly biased polar residue distribution toward the β-ionone ring, and (iv) tight association with the retinal chromophore to improve the retinal planarity, which appears essential for fast photocycle kinetics. Based on these molecular insights, we have generated ChrimsonSA (S169A, Super red-shifted and Accelerated) with more than 20 nm further red-shifted absorption, significantly reduced blue-light-sensitivity, and accelerated closing kinetics, compared to wild-type Chrimson. ChrimsonSA expressed well in hippocampal neurons and allowed spiking with red light while the prevalence of blue-light-evoked action potentials was strongly reduced, making it a useful tool for dual-color applications.

## Results

### Structural determination

**Fig. 1:**
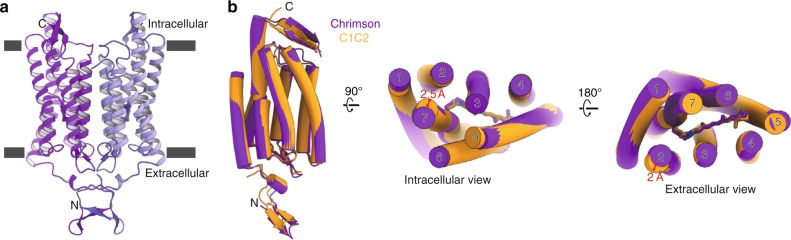
Overall structure of Chrimson. **a** The dimeric structure of Chrimson is shown in ribbon models, with the two protomers in different colors. **b** Chrimson (purple) and C1C2 (orange) are superimposed, viewed from within the membrane plane (left), the intracellular side (center) and the extracellular side (right). The TM numbering is indicated on each helix, and the deviation of the helix termini for TM2 and TM7 are indicated by red arrows

As the expression level of wild-type Chrimson is very low in insect cells, we tested two Chrimson variants in which the extracellular N-terminal sequence was replaced by either the corresponding sequence of the *Chloromonas subdivisa* channelrhodopsin (*Cs*Chrimson) or the N-terminal sequence of *Cr*ChR1 (C1Chrimson) (Supplementary Figure 1). Although CsChrimson showed improved expression and excellent stability in detergent, the purified protein yielded only poorly diffracting crystals, with low reproducibility. In contrast, C1Chrimson yielded crystals that diffracted X-rays to the maximum resolution of 2.6 Å, and the structure was determined by molecular replacement, using the C1C2 structure as the template (PDB: 3UG9) (Fig. 1a, Supplementary Figure 2 and Supplementary Table 1). The photocurrent properties of Chrimson were minimally affected by the different N-terminus, and red light activation, high proton selectivity, and fast pH-dependent photocurrent kinetics were preserved in all three Chrimson variants (Supplementary Figure 4a–e). For simplicity, we will therefore refer to C1Chrimson as Chrimson in the following sections.

### Gate interactions

Although Chrimson is distantly related to the *Cr*ChRs (about 50% sequence identity over transmembrane region) (Supplementary Figure 1), the overall structure of Chrimson superimposes well with those of C1C2 (Fig. 1b). The two protomers in the asymmetric unit form a dimer, with each protomer composed of the extracellular N-terminal domain, 7TM helix bundle and the C-terminal β-hairpin loop region. The *Cr*ChR1-derived N-terminal residues mediate the crystal packing contacts between the layers in the lipidic cubic phase crystal and facilitate the dimeric interaction by forming the inter-protomeric β-sheet and three disulfide bonds (Supplementary Figure 2b). While the outer membranous regions are partially disordered, the TM region is clearly visible in the electron density map, including the covalently attached all-trans retinal (Supplementary Figure 3). The structures of the two protomers (mol A and B) are essentially the same (RMSD = 0.66) (Supplementary Figure 2c), but due to their different crystal packing contacts, mol A presents better electron density. Therefore, our discussion below is based on the mol A structure of Chrimson. In direct superposition of Chrimson with C1C2 and *Cr*ChR2, we observed slight differences in the helix orientations, especially at TM2 and TM7, which deviate by about 2 Å on the extracellular side and 2.5 Å on the intracellular side, respectively (Fig. 1b). As these helices are directly involved in the ion pore formation in ChRs, by mediating conformational changes during the photocycle^21^, these variations might be partly responsible for the differences in ion permeation of Chrimson and *Cr*ChRs^22^.

**Fig. 2:**
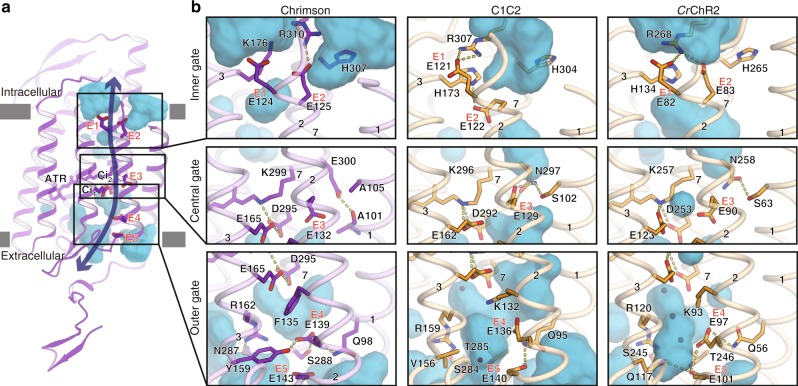
Ion pores of Chrimson and ChRs. **a** Water accessible cavities are illustrated in the Chrimson structure, with the putative ion pathway indicated by an arrow. Five glutamic acid residues lining the ion pore (E1–5) and two counterion residues (Ci_1_ and Ci_2_) are indicated by sticks, and the three constriction sites for the inner, central and outer gates are indicated by boxes. **b** Comparison of the constriction sites of Chrimson (left panels), C1C2 (center panels), and *Cr*ChR2 (right panels), for the inner (upper panels), central (middle panels), and outer (lower panels) gates. The constituent residues are shown as sticks, and the TM helix number is indicated on each helix

In Chrimson, the putative ion pathway, formed by TM2, 3, 6, and 7, is lined by five highly conserved negatively charged residues, Glu124 (E1), Glu125 (E2), Glu132 (E3), Glu139 (E4), and Glu143 (E5) (residue numbering is according to wild-type Chrimson^16^) (Fig. 2a). In the current ground state structure of Chrimson, the ion-conducting pore is closed by three constriction sites that restrict ion permeation, on the intracellular side (inner gate), at the middle of the membrane (central gate), and on the extracellular side (outer gate) (Fig. 2). While the inner and central gates are also observed in C1C2 and *Cr*ChR2^7,13^, their constituents differ in several aspects (Fig. 2 and Supplementary Figure 5). The inner gates of C1C2 and *Cr*ChR2 are mainly formed by ionic interactions between the acidic residues (E1 and E2) and the basic residues (His173 and Arg307 in C1C2 and His134 and Arg268 in *Cr*ChR2) (Fig. 2b upper panels). In Chrimson, this histidine is substituted with lysine (Lys176), and both Glu124 (E1) and Glu125 (E2) are oriented toward the cytoplasmic solvent, with only E2 providing an ionic interaction with Arg310. The central gate is located adjacent to the retinal Schiff base (Fig. 2b middle panels), where three hydrophilic residues (Ser102, Glu129 (E3) and Asn297 in C1C2, and Ser63, Glu90 (E3) and Asn258 in *Cr*ChR2) occupy the pore and thereby hinder ion permeation. In Chrimson, two of the three residues are substituted, resulting in a rather loose packing. In addition, Ala105 (serine in C1C2 and *Cr*ChR2) is no longer involved in the gate formation, and instead, Glu300 forms a hydrogen bond with the backbone carbonyl of Ala101, which is one helical turn below the involved residues in C1C2 and *Cr*ChR2. Finally, we observed remarkable structural diversity at the extracellular end of the ion-conducting pathway among all three ChR structures. In Chrimson, direct hydrogen bonding between Glu139, Tyr159, and Ser288 interconnects TM2, TM3, and TM7 and constitutes the outer gate constriction (Fig. 2b lower panels). In contrast, C1C2 has an open tunnel that allows water access to the middle of the pathway, and *Cr*ChR2 has an extended water-filled cavity (EC2), which is interrupted at the extracellular end of TM2 by hydrogen bonds between Glu101, Gln117 and Thr246 (EG: Extracellular gate, Supplementary Figure 5). Mutations of the three pore-lining glutamates involved in the interhelical hydrogen bonds in Chrimson, namely, Glu125 (E2) in the inner gate, Glu300 in the central gate and Glu139 (E4) in the outer gate, caused dramatic decelerations in the photocurrent kinetics^22^, whereas the homologous mutations of E2 and E4 in *Cr*ChR2 did not affect the channel kinetics^23^. Therefore, the observed variations in the gating interactions may account for the fast gating kinetics of Chrimson ^22^.

### Unique counterion configuration in Chrimson

**Fig. 3:**
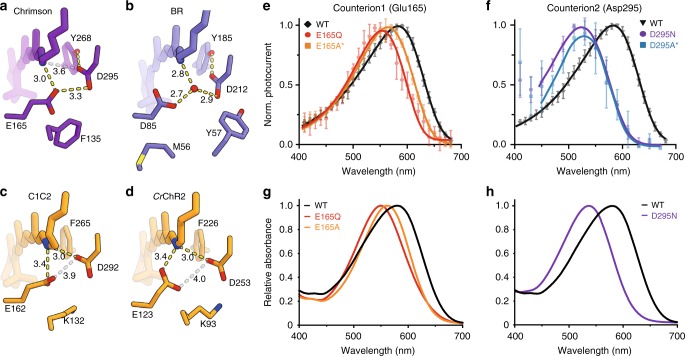
Counterion configuration of Chrimson and adjacent residues. **a**–**d** Hydrogen-bonding interactions around the protonated Schiff base are shown for Chrimson (**a**), BR (**b**), C1C2 (**c**), and *Cr*ChR2 (**d**). Proposed hydrogen-bonding interactions are indicated by yellow dotted lines, and additional possible interactions with longer distances are indicated by gray lines, with the distance (Å) indicated beside each line. **e**–**h** action (**e**, **f**) and absorption (**g**, **h**) spectra of the counterion residue mutants (Action spectra represent Mean ± SD; *n* = 5–7; symmetric 110 mM NaCl, pH_e,i_ 7.2 and −60 mV. Datasets indicated by asterisk are quoted from a previous study^22^. Same mutants are indicated by the same color codes. The fitted curves for the D295N and D295A action spectra were adjusted to the photocurrents activated by light of 440 nm or higher wavelength. Absorption spectrum of the D295A mutant could not be measured, due to its instability in the detergent solubilized form, and thus is not included in the panel (**h**)

The electrostatic interaction between the protonated Schiff base (PSB) and the counterion is the major determinant for the absorption spectrum of the retinal chromophore^21,24,25^. In general, negative charges in close proximity to the PSB stabilize the ground state and lead to a higher energy gap between the ground and excited states, resulting in blue-shifted absorption, while neutralization or spatial separation of the counterion charge leads to a lower energy gap and red-shifted absorption. In C1C2, the positive charge of the PSB is stabilized by two anionic counterion residues, Ci_1_ and Ci_2_ (Glu162 and Asp292, respectively), which are highly conserved among ChRs, including Chrimson (Glu165 and Asp295)^16,26^ (Fig. 3a–d). Recent studies have indicated that the highly red-shifted absorption of Chrimson is partly due to the protonation of the counterion residue Glu165^22,27^, which weakens the stabilization effect for the PSB, thus causing red-shifted absorption similar to that of the acidic form of BR (BR_605_)^28^. Consistent with these studies, the purified Chrimson protein showed a large pH-dependent spectrum shift (Supplementary Figure 6a and b). The crystals of Chrimson were obtained under low pH conditions (Methods), and the blue color of the crystals indicates that the current structure represents its red light-absorbing (protonated) state (Supplementary Figure 6c and d). Regarding the two counterion residues, Glu165 is in 3.0 Å proximity to the PSB, while Asp295 is farther away, at 3.6 Å (Fig. 3a). Glu165 resides just beside the PSB, a location corresponding to the hydrated water molecule in the bacteriorhodopsin (BR) dark state (Fig. 3b), and the two counterion residues Glu165 and Asp295 are only 3.3 Å apart from each other (Supplementary Figure 6e). Therefore, the Glu165 proton might be partly shared by both counterion residues, via hydrogen bond interactions. Most importantly, the counterion complex is shielded from the extracellular bulk solvent by the hydrophobic side chain of Phe135, which is essential to stabilize the protonated form of the carboxylate, as reported in previous studies^22,27,29^. (Fig. 3a and Supplementary Figure 6e and f). In contrast, in C1C2 and *Cr*ChR2, the phenylalanine is substituted with lysine (Lys132 in C1C2 and Lys93 in ChR2), and its positively charged side chain stabilizes the ionized (deprotonated) form of the Ci_1_ carboxylate (Glu162 in C1C2 and Glu123 in *Cr*ChR2) (Fig. 3c and d and Supplementary Figure 6g).

### Outer gate and ion pore of Chrimson

As the counterion configuration of Chrimson is essentially stabilized by the carboxylic acids of both Glu165 and Asp295, the mutation of either residue caused a blue-shift in the action and absorption peaks by 5–60 nm and abolished the pH dependence (Fig. 3e, f and Supplementary Figure 8a–c). This can be explained by the decreased *p*K_a_ and consequent deprotonation of the remaining counterion residue (Asp295 for the Glu165 mutants and Glu165 for the Asp295 mutants), consistent with previous studies of Chrimson^22,27^ and other channelrhodopsins^29^. The blue-shift was more prominent when Asp295 is mutated, whereas the mutation of Glu165 to Gln or Ala had a moderate effect. This is also consistent with the counterion configuration in the current structure. Since the PSB is closer to Glu165 than to Asp295, the neutralization of Asp295 should focus the negative charge exclusively on Glu165, which is much closer to the PSB, thus causing a larger blue-shift. Whereas blue-shift was equally pronounced for all Asp295 mutants, it varied largely for different mutants of Glu165 (Fig. 3e, h and Supplementary Figure 8d) as the introduced side chain might affect position and *p*K_a_ of the remaining counterion residue Asp295.

**Fig. 4:**
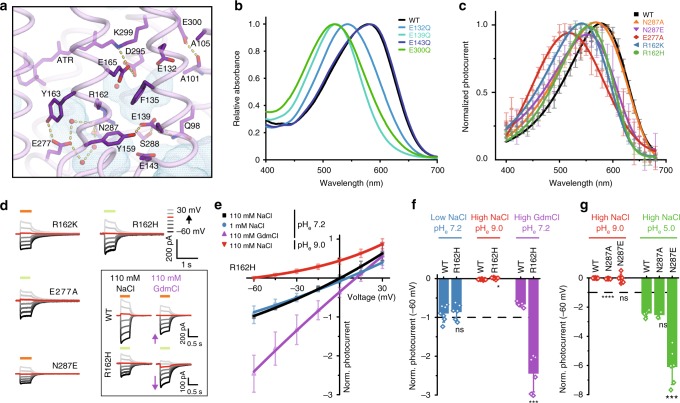
Outer gate and the extracellular ion pore. **a** Residues around the outer gate are shown as sticks, with the interactions indicated by yellow lines. Water accessible cavities are indicated by blue meshes. **b** Absorption spectra of the carboxylate mutants within the extracellular ion pore and the outer gate. **c** Mutations in the extracellular ion pore caused blue-shifting of the action spectrum. Normalized peak photocurrents after 10 ms excitation at different wavelengths of equal photon count (Mean ± SD; *n* = 5–7; symmetric 110 mM NaCl, pH_e,i_ 7.2 and −60 mV) are shown. **d** Representative photocurrent traces of the Arg162 and Asn287 mutants at different voltages illuminated either with 550 nm light (green line) or with 580 nm light (orange line) depending on peak absorption. The inset shows representative photocurrent traces of wild type (upper) and R162H (bottom) upon replacement of Na^+^ with guanidinium. The purple arrows indicate the photocurrent increase or decrease upon cation exchange in the R162H mutant and Chrimson WT, respectively. **e** Current-voltage dependence of normalized R162H photocurrents in extracellular solutions of different cation or proton composition (mean ± SD; *n* = 6–9; intracellular 110 mM NaCl, pH_i_ 7.2). R162H is still selective for protons, as the current-voltage dependence drastically shifts under the alkaline conditions (two-sample *t*-test with *p* = 0.025), while it is not affected by decreasing the Na^+^ ion concentration (*p* = 0.75). In contrast, the current-voltage dependence is drastically shifted when Na^+^ is replaced by guanidinium (*p* = 0.0002), indicating that R162H is permeable to guanidinium. **f** Normalized photocurrents at the ionic conditions of (**e**) and −60 mV compared to WT (Mean ± SD, *n* = 5–10; intracellular 110 mM NaCl, pH_i_ 7.2, junction potential corrected). **g** Normalized photocurrent amplitudes of WT and N287 mutants at different extracellular pH (Mean ± SD; *n* = 2–10; intracellular 110 mM NaCl, pH_i_ 7.2; −60 mV). Photocurrents of both mutants and WT were equally reduced at low proton concentration indicating comparable high proton selectivity (two-sample *t*-test compared to WT with *p* < 0.0001 for N287A and *p* = 0.9 for N287E). At extracellular pH 5.0 photocurrent increase of the N287E mutant was significantly higher than for WT or the N287A mutant (*p* = 0.5 for N287A and *p* = 0.0005 for N287E)

In addition to the counterion residues, mutations of the neighboring carboxylates, such as Glu132 (E3), Glu139 (E4), and Glu300, located within the extracellular cavity, also led to strong blue-shifts (Fig. 4a, b and Supplementary Figure 7d–f), although these residues are not in the proximity of the Schiff base. The effects of these mutants are more prominent, as compared to the counterion mutants (E165Q and D295N), although these mutants still retained the pH-dependency and showed small fractions of red-light sensitive components under the low pH conditions (Supplementary Figure 7d–f). Therefore, we conclude that both mutations predominantly affect the protonation of the counterion residues, via either the hydrogen-bonding network or long-range electrostatic interactions. In contrast, the mutation of Glu143, located outside the extracellular cavity, had minimal effects on the absorption spectrum and the pH-dependent shift (Supplementary Figure 7g). These results indicated that the cluster of carboxylate residues that are separated from the extracellular solvent by the outer gate might also contribute to the protonation of the counterion residues. Consistently, mutations that destabilize the outer gate hydrogen-bonding network, such as Tyr159 and Glu139 (E4), caused a largely blue-shifted activation spectrum^22^.

We further investigated the residues constituting the extracellular ion-conducting pathway. At the exit of this pathway, Arg162 partly participates in the extracellular ion pore, together with Tyr159 and Phe135 (Fig. 4a). Stabilized by a hydrogen bond with Asn287, Arg162 is slightly oriented toward the negatively charged residue Glu277, which is exposed to the solvent. The mutation of Arg162, as well as those of Asn287 and Glu277, caused a large blue-shift of the activation spectrum (Fig. 4c), indicating that Arg162 electrically affects the PSB environment, and its precise arrangement is important for the protonation of Glu165. By analogy to its functional significance in other microbial rhodopsins^30^, we assumed that Arg162 is directly involved in channel opening and thus characterized its role in more detail. Whereas the R162A mutant is completely non-functional, as previously shown in C1C2 and *Cr*ChR2^7,31^, mutations to positively charged residues preserved the channel function, although the mutants showed significantly reduced amplitudes and impaired kinetics (Fig. 4d–f and Supplementary Figure 8a–d). Unexpectedly, a photocurrent analysis in HEK cells indicated that the ion-conducting pore of the R162H mutant is permeable to the large cation guanidinium, which is only poorly conducted by wild-type Chrimson (Fig. 4e)^22^. Furthermore, the photocurrent amplitudes were significantly reduced by the N287E mutation, which may stabilize the Arg162 orientation in the current dark state structure, whereas the photocurrents were partly restored in pH 5, where this effect should be weakened (Fig. 4d, g and Supplementary Figure 8b and c). As previous studies have shown that two neighboring carboxylate residues, E4 (Glu139) and E5 (Glu143), located at the extracellular end of TM2, are responsible for the proton selectivity in Chrimson^22^, these results are in support of Arg162 being directly involved in the formation of the ion-conducting pore and possibly contributing to the ion selective filter of Chrimson.

### Retinal binding pocket

**Fig. 5:**
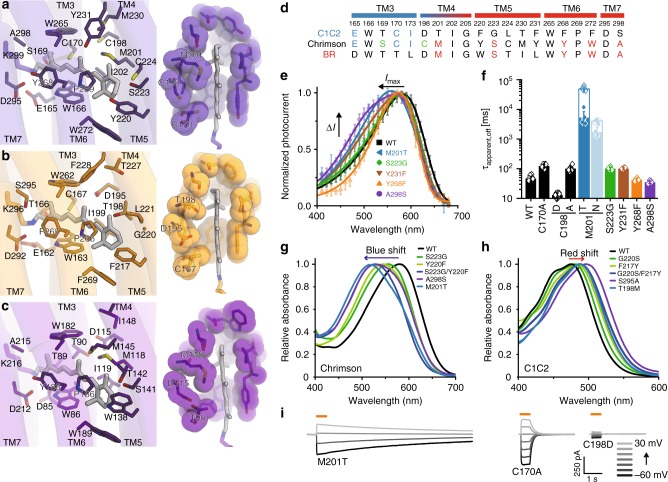
Retinal binding pocket. **a**–**c** Structural comparisons of the retinal binding pockets of Chrimson (**a**), C1C2 (**b**), and BR (**c**). As the retinal pockets of C1C2 and *Cr*ChR2 are quite similar, C1C2 is shown as the representative structure of the *Cr*ChRs. Residues constituting retinal binding pocket are shown in sticks (left panels). CPK model representations shows different association in the retinal binding pocket (right panels). **d** Sequence comparison of Chrimson with C1C2 and BR. The residue numbering of Chrimson is indicated above the sequence. Chrimson resembles BR in TM5–7, surrounding the polyene chain and β-ionone ring, while it resembles C1C2 (or *Cr*ChRs) in TM3, located near the ion-conducting pore. Residues common to BR and C1C2 are depicted in cyan and red, respectively. **e** Mutations that affect action spectrum. Normalized peak photocurrents after 10 ms excitation at different wavelengths of equal photon count (Mean ± SD; *n* = 5–8; symmetric 110 mM NaCl, pH_e,i_ 7.2 and −60 mV) are shown. **f** Apparent photocurrent off-kinetics of retinal binding pocket mutants (τ_apparent off_; Mean ± SD; *n* = 5–11; symmetric 110 mM NaCl, pH_e,i_ 7.2 and −60 mV). Empty columns for M201T and M201N represent conductance measurements by short 20 ms voltage pulses to −60 mV at 0.2 and 0.5 Hz, respectively, and at a holding potential of 0 mV in order to reduce kinetic artefacts imposed by intracellular acidification due to continuous proton influx as previously reported^22^ (Mean ± SD; *n* = 5–6). **g**, **h** Absorption spectra of wild type and mutant Chrimson (**g**) and C1C2 (**h**). Mutations at the same positions are indicated by the same color codes. Peak shifts caused by mutation is indicated by blue or red arrow. **i** Representative photocurrent traces of the M201T, C170A, and C198D mutants, measured at different voltages in symmetric 110 mM NaCl, pH_e,i_ 7.2 and illuminated with 580 nm light (orange line)

As described above, the outer gate and the consequential protonation of the counterion residues essentially contribute to the red-shifted absorption of Chrimson. However, even at high pH with a deprotonated counterion, Chrimson absorbs a longer wavelength (530 nm) than C1C2 (480 nm), implying an additional mechanism contributing to the large red-shifted absorption. Compared to C1C2, about half of the residues constituting the retinal binding pocket are substituted in Chrimson, and the structural comparison revealed that Chrimson resembles BR rather than C1C2, with respect to the residues surrounding the polyene chain and the β-ionone ring (Fig. 5a–d). We constructed Chrimson mutants with residues mutated to the corresponding residues of C1C2, and measured their action spectra (Fig. 5e, f and Supplementary Figure 8). The spectra of M201T, S223G, Y231F, Y268F, and A298S were all blue-shifted by 10–30 nm as compared to the wild type, indicating that all five residues collectively contribute to the red-shifted activation of Chrimson. According to the general mechanism of color tuning in retinal proteins, negative polarity near the β-ionone ring stabilizes a light-excited intermediate that involves charge movement toward the β-ionone ring during retinal isomerization and thus leads to the red-shifted absorption, while negative polarity near the Schiff base leads to the blue-shifted absorption^25^. Consistently, polar residues such as Ser223 and Tyr231 are located near the β-ionone ring, while the non-polar residue Ala298 is located near the Schiff base (Fig. 5a). All of these residues are conserved in other red-shifted variants such as *V*ChR1 and ReaChR, but they are substituted with the non-polar residues Gly220 and Phe228, and the polar residue Ser295, respectively, in C1C2 (Fig. 5b and Supplementary Figure 1). The contributions of these residues to the red-shifted absorption were further confirmed by measuring the absorption spectra of the above mutants (S223G, Y231F, and A298S) (Fig. 5g), which though showed slightly larger blue-shifts as compared to the action spectra. Conversely, the introduction of these residues to C1C2 (G220S, F217Y, and S295A) caused a red-shift of the absorption peak by about 10–30 nm (Fig. 5h). These results show that the biased distribution of the polar residues throughout the retinal binding pocket contributes to the red-shifted absorption of Chrimson and other red-shifted variants. Meanwhile, Tyr268 is the exception, as the non-polar substitution Y268F caused blue-shifted absorption by about 10 nm, although it is located near the Schiff base (Fig. 5e). As Tyr268 is engaged in the hydrogen bond with the counterion residue Asp295 (Figs. 3a and 5a), the mutation to phenylalanine probably allows Asp295 to approach toward the PSB and stabilizes its positive charge, thus causing the blue-shift.

Additional structural differences were found in TM4 and TM5, which flank the retinal binding pocket (Fig. 5a–c right panels). Chrimson has several bulky residues on these helices. In particular, the bulky side chain of Met201 tightly packs against the polyene chain of retinal (Fig. 5a). As previously shown for C1C2 and archaerhodopsin-3, the residue at this position is particularly important for the torsion angle at the C6 = C7 bond^8^. In agreement with this study, the mutation of Met201 to the smaller residue, threonine, caused a large blue-shift in the absorption peak by about 50–80 nm (Fig. 5g and Supplementary Figure 7h). In C1C2, the reverse mutation T198M caused a red-shifted absorption (Fig. 5h). These observations suggest that tight packing against the polyene chain is important for the red-shifted absorption, probably by affecting the π-electron conjugation.

Remarkably, the M201T mutant further showed extremely slow closing kinetics, and photocurrents were sustained for minutes (Fig. 5f, i), indicating an additional important role of Met201 in channel gating kinetics. Also in bacteriorhodopsin, the alanine mutation of the corresponding methionine (Met118) or the adjacent methionine (Met145) leads to a slower photocycle and a consequent reduction in the proton pump activity, as well as a large blue-shift in the absorption^32,33^. These results indicate that the structural rigidity around the β-ionone ring is especially crucial for the proper photocycle process. Therefore, the slow kinetics of the M201T mutant is probably associated with the loose interaction at the retinal binding pocket, which decelerates conformational propagation from the isomerized retinal to the protein. The effect of the M201T mutation is reminiscent of the step function opsin (SFO) mutants of the *Cr*ChRs, in which the mutation of the DC-pair (Asp195 and Cys167 in C1C2 and Asp156 and Cys128 in *Cr*ChR2) caused extremely slow channel kinetics, by affecting proton exchange reaction at the Schiff base^34–36^. Although Met201 occupies a position close to the DC-pair in *Cr*ChRs (Fig. 5a and b), the Asp residue of DC-pair, which is the putative internal proton donor for the deprotonated Schiff base in *Cr*ChR2^37^, is substituted with cysteine (Cys198), suggesting a completely different mechanism between the Chrimson M201T mutant and the *Cr*ChR SFO mutants. In Chrimson, mutation of the Cys128 homolog (C170A) had minimal impact on the photocurrent kinetics, and the reintroduction of the DC-pair (C198D) caused nearly complete loss of the photocurrent, albeit accelerated photocurrent kinetics (Fig. 5f), further supporting a different gating mechanism of Chrimson compared to the previously proposed gating model for *Cr*ChRs. The observation in Chrimson is more in agreement with the general importance of the retinal binding pocket stability for the overall photocycle^38^, which is indeed different between Chrimson and *Cr*ChRs (Fig. 5a, b). This notion is also consistent with the recent study showing that mutations near the β-ionone ring (TM6) affect the channel kinetics in Chrimson and other ChRs^39^.

### Rational design of a more red-shifted Chrimson

**Fig. 6:**
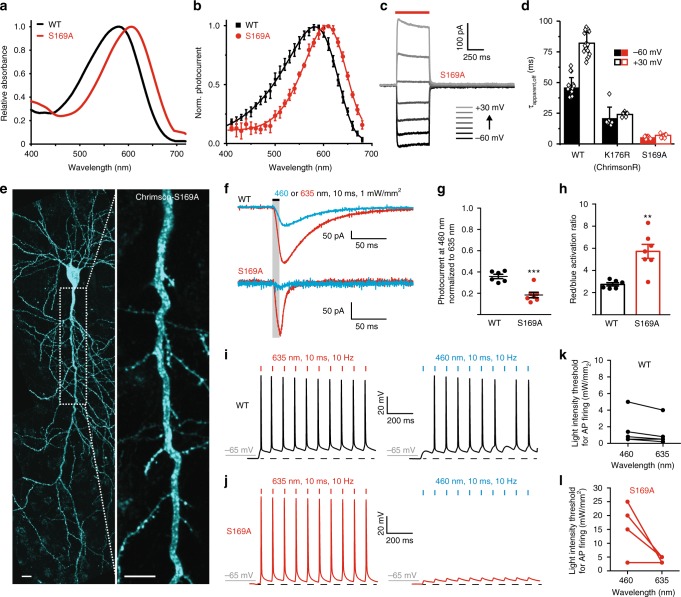
Chrimson-S169A in rat CA1 pyramidal cells. **a**, **b** Red-shifted absorption spectrum (**a**) and action spectrum (**b**) of Chrimson S169A (ChrimsonSA) (Mean ± SD; *n* = 7; 10 ms illumination of equal photon counts, symmetric 110 mM NaCl, pH_e,i_ 7.2 and −60 mV). **c**, **d** Photocurrents of ChrimsonSA in HEK cells. Representative photocurrent traces (**c**) and channel closing kinetics (**d**) at the same ionic conditions as in (**b**) are shown. **e** CA1 pyramidal neuron heterogeneously expressing ChrimsonSA-mCerulean, 5 days after electroporation (stitched maximum intensity projections of two-photon images). The inset shows a magnified view of the apical dendrite. The scale bars indicate 10 μm. **f** Representative photocurrent traces of Chrimson-WT (top) and ChrimsonSA (bottom) expressing CA1 pyramidal cells evoked with either 635 nm (red trace) or 460 nm (blue trace, 1 mW/ mm^2^) light. The ChrimsonSA traces were scaled to WT Chrimson **g** Relative photocurrent responses of WT Chrimson and ChrimsonSA to blue light (460 nm, 10 ms, 1 mW/ mm^2^). Values were normalized to the response to red light (635 nm, 10 ms, 1 mW/ mm^2^). Dots represent single cells. Lines show mean ± SEM (n_WT_ = 6; n_S169A_ = 7). **h** Quantification of the red/blue activation ratio. Bar plots show mean ± SEM. **i**, **j** Action potentials triggered by red light pulses at a frequency of 10 Hz in cells expressing WT Chrimson (**i**) and ChrimsonSA (**j**) at threshold light intensities of 0.5 and 5 mW/mm^2^, respectively. Using the same light intensities, the action potentials were triggered by blue light in WT, but not in the ChrimsonSA. Dashed lines represent the membrane resting potential. **k**, **l** The light intensities at 460 nm and 635 nm required to reach the action potential threshold in neurons expressing WT Chrimson (**k**) or ChrimsonSA (**l**)

The current structure and concomitant extensive mutation analysis revealed three important factors underlying the large red-shift of Chrimson: the unique protonation state of the counterion residues, the highly biased distribution of the polar residues toward the β-ionone ring, and the structural rigidity of the retinal binding pocket. These notions allowed us to rationally design a more red-shifted Chrimson variant. As two of the three factors, the electrostatic stabilization of the PSB and the tight association around the retinal pocket, are already highly optimized in Chrimson, we engineered the polarity distribution around the retinal binding pocket and successfully obtained a red-shifted mutant by decreasing the polarity near the Schiff base (S169A) (Fig. 5a). The absorption and action spectra peaks of this mutant are at 608 nm and 605 nm, respectively, which are red-shifted by about 20 nm as compared to wild-type Chrimson (Fig. 6a, b and Supplementary Figure 9a and b). Surprisingly, this mutant displayed 10-fold accelerated channel closing kinetics, as compared to wild-type Chrimson (Fig. 6c), which is comparable to the recently reported engineered Chrimson variants with fast closing kinetics (f-Chrimson and vf-Chrimson)^39^. Due to the red-shift and the accelerated closing kinetics, we named this new mutant ChrimsonSA (S169A, Super red-shifted and Accelerated). To validate its usefulness for optogenetic applications, we tested ChrimsonSA in hippocampal neurons (Fig. 6e–l). Although the photocurrent density of the S169A mutant was reduced in HEK cells, as compared to wild-type Chrimson (12 ± 4 pA/pF for S169A versus 90 ± 60 pA/pF for WT) (Supplementary Fig. 8b), its photocurrent size is comparable to the *Cr*ChR2 stationary currents measured in HEK cells under the same conditions (15 ± 7 pA/pF)^22^. In neurons, photocurrents of ChrimsonSA were smaller than WT Chrimson but sufficient to trigger action potential firing with 635 nm light at intensities of 5 mW/mm^2^ or less (Fig. 6l), confirming its utility for optogenetic applications. Neurons expressing ChrimsonSA further showed significantly smaller blue-light-evoked photocurrents, as compared to red light, than neurons expressing wild-type Chrimson, resulting in a higher red/blue-light-activation ratio (Fig. 6f–h). As a consequence, the action potentials were selectively evoked by red light, with a significantly decreased probability of blue-light-evoked action potential firing at similar intensities (Fig. 6i–l). Furthermore, due to the accelerated channel closing kinetics of this mutant, the recovery of the membrane potential after each spike was quicker as compared to neurons expressing wild-type Chrimson, leading to a more natural waveform of light-evoked action potentials (Supplementary Fig. 9c, d). Thus, ChrimsonSA is a useful optogenetic tool for neuronal spiking with far-red light and therefore of high value for dual-color optogenetic experiments.

## Discussion

Chrimson is widely used in optogenetic experiments, since its red-shifted action spectrum allows deep tissue penetration of the excitation light, activation in a spectral range beyond the natural sensitivity of native photoreceptors in many animal species and combination with blue-light-activated tools for dual-color experiments. In the present study, we have reported the crystal structure of Chrimson, which revealed substitutions and structural differences in the putative ion pore, as compared to the *Cr*ChRs, which contribute to the different gating kinetics and the elevated proton selectivity of Chrimson. While the putative ion pore shares similarity to other ChRs, the structural motifs of Chrimson around the retinal binding pocket are more similar to those of BR. Since ChRs presumably evolved from prokaryotic light-driven pumps^40^, Chrimson might therefore reflect an early branching of ChRs during the molecular evolution from its prokaryotic ancestors. We conducted an extensive mutational analysis, which revealed the structural features in the counterion region and retinal binding pocket that enable the red light absorption and fast channel gating transitions in Chrimson. Based on these findings, we engineered ChrimsonSA, with further red-shifted peak absorption beyond 600 nm and strongly accelerated photocurrent closing kinetics. ChrimsonSA represents the most red-shifted microbial rhodopsin known to date that preserves protein function. Due to its reduced blue-light sensitivity, it is an excellent optogenetic actuator for dual-color applications.

## Methods

### Expression and purification of Chrimson

The C1Chrimson was constructed by replacing the N-terminal sequence (1–79) of *Cs*Chrimson with that of *Clamydomonas reinhardtii Cr*ChR1(1–76), by In-Fusion Cloning Kit (Clontech). The construct was subcloned into a modified pFastBac1 vector for expression in *Sf9* insect cells (11496015, Thermo Fischer Scientific), with the tobacco etch virus (TEV) protease cleavage site, the enhanced GFP (EGFP), and the FLAG-tag (EGFP-FLAG) fused at the C-terminus. Baculovirus-infected *Sf*9 cells were cultured in Sf900II (Invitrogen) at 27 °C for 24 h, and 100 µl all-trans retinal (SIGMA-ALDRICH) was added at 24 h post-infection. Cells were harvested 48 h after infection by centrifugation at 6000× *g* for 10 min. The pellets were disrupted by sonication and resuspended in buffer containing 150 mM NaCl, 50 mM HEPES, pH 7.0, 5% glycerol and 0.1 mM phenylmethylsulfonyl fluoride (PMSF). The cell debris was cleared by centrifugation at 10,000× *g* for 30 min, and the crude membrane fraction was collected by ultracentrifugation (Ti45 rotor, 215,000× *g*, 1 h). This fraction was solubilized in buffer containing 150 mM NaCl, 50 mM HEPES, pH 7.0, 5% glycerol, 0.1 mM PMSF, 2.5% *n*-dodecyl-β-D-maltoside (DDM), and 0.5% cholesteryl hemisuccinate (CHS). The insoluble material was removed by ultracentrifugation (Ti70 rotor, 208,000× *g*, 30 min), and the supernatant was mixed with ANTI-FLAG M2 Agarose Affinity Gel(SIGMA-ALDRICH). After binding for 1.5 h, the flow-through was discarded. Following the cleavage of EGFP–FLAG by TEV protease (homemade), the flow-through containing Chrimson was collected, concentrated, and further purified by size-exclusion chromatography in 150 mM NaCl, 50mM HEPES, pH 7.0, 5% glycerol, 0.05% DDM, and 0.01% CHS. Peak fractions were pooled and concentrated to 7.0 mg/ml for crystallization.

### Crystallization

The purified C1Chrimson protein was mixed with monoolein (SIGMA-ALDICH) in a 2:3 protein to lipid ratio (w/w). Aliquots (50 nl) of the protein-LCP mixture were spotted on a 96-well sitting-drop plate and overlaid with 800 μl of precipitant solution by the crystallization robot, Crystal Gryphon (Art Robbins Instruments). Crystals were obtained in 28–33% (w/v) PEG500DME, 100 mM Na citrate, pH 5.0, 100 mM Na malonate, pH 7.0, and 10–40 mM sarcosine. All crystals were incubated for 4 weeks in the dark. The crystals were harvested using micromounts (MiTeGen) and flash-cooled in liquid nitrogen without any additional cryoprotectant.

### Structure determination

X-ray diffraction datasets were collected at the SPring-8 BL32XU beamline. Data processing and merging of multiple crystals were performed using the program KAMO (https://github.com/keitaroyam/yamtbx/blob/master/doc/kamo-en.md)^41^. Each dataset was indexed and integrated using XDS^42^. Since the crystals were anisotropic, with varied lattice parameters, datasets with a *c*-axis less than 173 Å and a *b*-axis less than 82 Å were selected. Finally a group of outlier-rejected datasets was scaled and merged using XSCALE. The structure was solved by the molecular replacement method implemented in Phenix Phaser-MR^43^, using the model of C1C2 (PDB: 3UG9). The structure was manually modified to fit into the electron density maps, using the program Coot^44^, and then refined with the programs Phenix^43^ and Refmac5 in the CCP4 suite^45^. Figures were prepared with Cuemol (http://www.cuemol.org). Protein internal cavities are analyzed by the program hollow^46^.

### Ultraviolet/visible spectroscopy

C1Chrimson was fused with the C-terminal FLAG-tag sequence, and expression and purification were performed as described above. The peak fraction of the size-exclusion chromatography was collected and concentrated for the measurement. The pH was adjusted by the addition of 20 μl 1 M buffer (pH5:Na-acetate, pH6:Na-citrate, pH7:HEPES-NaOH, pH8:Tricine-NaOH, pH9:Tris-HCl) to 100 μl of the protein solution. Ultraviolet/visible absorption spectra were recorded with an Ultrospec 3300 Pro ultraviolet/visible spectrophotometer (Amersham Biosciences), using 1-cm quartz cuvettes.

### HEK-cell electrophysiology

The coding sequences of Chrimson (KF992060.1), CsChrimson (KJ995863.2), and C1Chrimson were cloned into the pmCerulean-C1 vector (using the NheI and AgeI restriction sites for Chrimson and CsChrimson or the Nhe1 and Kpn2I restriction sites for C1Chrimson) and expressed under the control of the CMV-promotor. Site-directed mutagenesis was performed using a QuickChange Site-Directed Mutagenesis Kit (Agilent Technologies, Santa Clara, CA), according to the manufacturer’s instructions. As the Chrimson photocurrents were nearly unaffected by the targeting strategy (Supplementary Figure 4), we employed CsChrimson as the backbone for the electrophysiological mutant analysis.

HEK cell preparation and whole-cell patch-clamp experiments were performed as previously described^22,28^. HEK293 (human embryonic kidney) cells (85120602, Sigma-Aldrich) were cultured in Dulbecco’s Modified Medium (DMEM) with stable glutamine (Biochrom, Berlin, Germany) supplemented with 10% (v/v) fetal bovine serum (FBS Superior; Biochrom, Berlin, Germany), 1 μM all-trans retinal and 100 µg/ml penicillin/streptomycin (Biochrom, Berlin, Germany). The cells were seeded on coverslips at a concentration of 0.75 × 10^5^ cells/ml and transiently transfected using the FuGENE® HD Transfection Reagent (Promega, Madison, WI) 28–48 h before measurement.

Patch pipettes were prepared from borosilicate glass capillaries (G150F-3; Warner Instruments, Hamden, CT) using a P-1000 micropipette puller (Sutter Instruments, Novato, CA) and were subsequently fire polished. The pipette resistance was between 1.8 and 3.0 MΩ. Single fluorescent cells were identified using an Axiovert 100 inverted microscope (Carl Zeiss, Jena, Germany). Monochromatic light (±7 nm) was provided by a Polychrome V monochromator (TILL Photonics, Planegg, Germany), attenuated by a motorized neutral density filter wheel (Newport, Irvine, CA) for equal photon fluxes at different excitation wavelengths, and temporally controlled by a VS25 and VCM-D1 shutter system (Vincent Associates, Rochester, NY). Recorded signals were filtered at 2 kHz using an AxoPatch 200B amplifier (Molecular Devices, Sunnyvale, CA) and digitized using a DigiData 1440 A digitizer (Molecular Devices, Sunnyvale, CA) at a sampling rate of 5–10 kHz. The reference bath electrode was connected to the bath solution via a 140 mM NaCl agar bridge. The extracellular buffer exchange was performed manually by adding at least 5 ml of the respective buffer to the recording chamber (500 µl chamber volume) while a Ringer Bath Handler MPCU (Lorenz Messgerätebau, Katlenburg-Lindau, Germany) maintained a constant bath level. Standard bath solutions contained 110 mM NaCl, 1 mM KCl, 1 mM CsCl, 2 mM CaCl_2_, 2 mM MgCl_2_, and 10 mM HEPES at pH_e_ 7.2 (with glucose added up to 310 mOsm). Standard pipette solution contained 110 mM NaCl, 1 mM KCl, 1 mM CsCl, 2 mM CaCl_2_, 2 mM MgCl_2_, 10 mM EGTA, and 10 mM HEPES at pH_i_ 7.2 (glucose added up to 290 mOsm). For ion selectivity measurements, NaCl was replaced by either 110 mM GuanidiniumCl or 110 mM NMDGCl with 1 mM NaCl remaining, and HEPES was substituted with citric acid or TRIS for pH 5 or pH 9 respectively. For the measurement of ultra slow closing kinetics (empty columns Fig. 5f) cells were clamped at 0 mV and channel conductance was probed by short 20 ms voltage pulses to −60 mV at the slow frequency of 0.2 or 0.5 Hz (for M201T or M201N, respectively) in order to reduce kinetic artefacts imposed by intracellular acidification due to continuous proton influx as previously reported^22^. The light intensities were measured after passing through all of the optics using a P9710 optometer (Gigahertz-Optik, Türkenfeld, Germany) and normalized to the water Plan-Apochromat 40 × /1.0 differential interference contrast (DIC) objective illuminated field (0.066 mm^2^). The maximum light intensity was 2.28 mW × mm^−2^ at 580 nm and 2.47 mW × mm^−2^ at 530 nm. All electrical recordings were controlled by the pCLAMP™ software (Molecular Devices, Sunnyvale, CA). The whole-cell recordings had a minimum membrane resistance of 500 MΩ (usual > 1 GΩ) and an access resistance below 10 MΩ.

Electrical recordings were analyzed using the Clampfit 10.7 software (Molecular Devices, Sunnyvale, CA), Microsoft Excel and Origin 2017® (OriginLab, Northampton, MA). Photocurrent traces were baseline corrected, filtered, and reduced in size for display purposes. Photocurrents were normalized to peak photocurrents at −60 mV under standard conditions of symmetric 110 mM NaCl pH_e/i_ 7.2. Action spectra were fitted using a parametric Weibull function (*y* = y0 + A*((w2–1)/w2)^((1-w2)/w2)*S^(w2–1)*exp(-s^w2 + (w2–1)/w2) with *S* = (x-xc)/w1 + ((w2–1)/w2)^(1/w2) and the estimated parameters *A*, y0, w1, w2, and xc). The photocurrent kinetics were estimated by biexponential fits and simplified by an apparent time constant (*τ*_apparent_) calculated as (*A*_1_*τ_1_ + *A*_2_*τ_2_)/(*A*_1_ + *A*_2_). The exact number of biological replicates for each measurement is provided in the figure legend. To compare the data, we performed two-sample *t*-tests with Welch’s correction in Origin 2017®. The significance thresholds were set at *p* < 0.05 (*), *p* < 0.01 (**), *p* < 0.001 (***), and *p* < 0.0001 (****).

### Neuronal recordings in hippocampal slice cultures

Chrimson wild type and the ChrimsonSA mutant were subcloned into neuron-specific expression vectors (pAAV backbone, human *synapsin* promoter). Organotypic slice cultures of rat hippocampus were prepared as described^14^ and transfected by single-cell electroporation^15^ after 14 days in vitro (DIV). Animal procedures were in accordance with the guidelines of local authorities and Directive 2010/63/EU. Plasmids were each diluted to 1 ng/μl in K-gluconate–based solution consisting of (in mM): 135 K-gluconate, 4 MgCl_2_, 4 Na_2_-ATP, 0.4 Na-GTP, 10 Na_2_-phosphocreatine, 3 ascorbate, 0.02 Alexa Fluor 594, and 10 HEPES (pH 7.2). An Axoporator 800 A (Molecular Devices) was used to deliver 50 hyperpolarizing pulses (−12 mV, 0.5 ms) at 50 Hz. At DIV 18–20, targeted patch-clamp recordings of transfected neurons were performed under visual guidance using a BX-51WI microscope (Olympus, Hamburg, Germany) equipped with Dodt-gradient contrast and a Double IPA integrated patch amplifier controlled with SutterPatch software (Sutter Instrument, Novato, CA, USA). Patch pipettes with a tip resistance of 3–4 MΩ were filled with (in mM): 135 K-gluconate, 4 MgCl_2_, 4 Na_2_-ATP, 0.4 Na-GTP, 10 Na_2_-phosphocreatine, 3 ascorbate, 0.2 EGTA, and 10 HEPES (pH 7.2). Artificial cerebrospinal fluid (ACSF) consisted of (in mM): 135 NaCl, 2.5 KCl, 2 CaCl_2_, 1 MgCl_2_, 10 Na-HEPES, 12.5 D-glucose, 1.25 NaH_2_PO_4_ (pH 7.4). Synaptic currents were blocked with 10 µM CPPene, 10 µM NBQX, and 100 µM picrotoxin (Tocris, Bristol, UK). Measurements were not liquid junction potential (LJP) corrected. The LJP was -15.5 mV. A 16-channel pE-4000 LED light engine (CoolLED, Andover, UK) was used for epifluorescence excitation and delivery of light pulses (ranging from 365 to 660 nm). Light intensity was measured in the object plane with a 1918-R power meter equipped with a calibrated 818-ST2-UV/D detector (Newport, Irvine CA) and divided by the illuminated field (0.134 mm^2^) of the LUMPLFLN 60XW objective (Olympus).

### Two-photon microscopy

The custom-built two-photon imaging setup was based on an Olympus BX-51WI upright microscope upgraded with a multiphoton imaging package (DF-Scope, Sutter Instrument, Novato, CA, USA and Rapp OptoElectronic, Wedel, Germany), and controlled by ScanImage (Vidrio Technologies, Ashburn, VA, USA). Fluorescence was detected through the objective (LUMPLFLN 60XW, Olympus, Hamburg, Germany) and the oil-immersion condenser (1.4 NA) using GaAsP-PMTs (Hamamatsu Photonics, Hamamatsu, Japan). A tuneable Ti:Sapphire laser (Chameleon Vision-S, Coherent, Dieburg, Germany) was set to 810 nm to excite cerulean.

## Electronic supplementary material

Supplementary Information

Peer Review File

Description of Additional Supplementary Files

Supplementary Data 1

## Data Availability

Data supporting the findings of this manuscript are available from the corresponding authors upon reasonable request. Coordinates and structure factors are deposited in the Protein Data Bank with accession codes PDB 5ZIH. Raw diffraction images are also deposited to Zenodo data repository (10.5281/zenodo.1319974).
