# Supplementary material for: Crystal structure of the red light-activated channelrhodopsin Chrimson
Source: Nat Commun. 2018 Sep 26;9:3949. doi: 10.1038/s41467-018-06421-9 (PMC6158191; doi:10.1038/s41467-018-06421-9)
Supplement: Supplementary file 1 — Supplementary Information [file 41467_2018_6421_MOESM1_ESM.pdf]

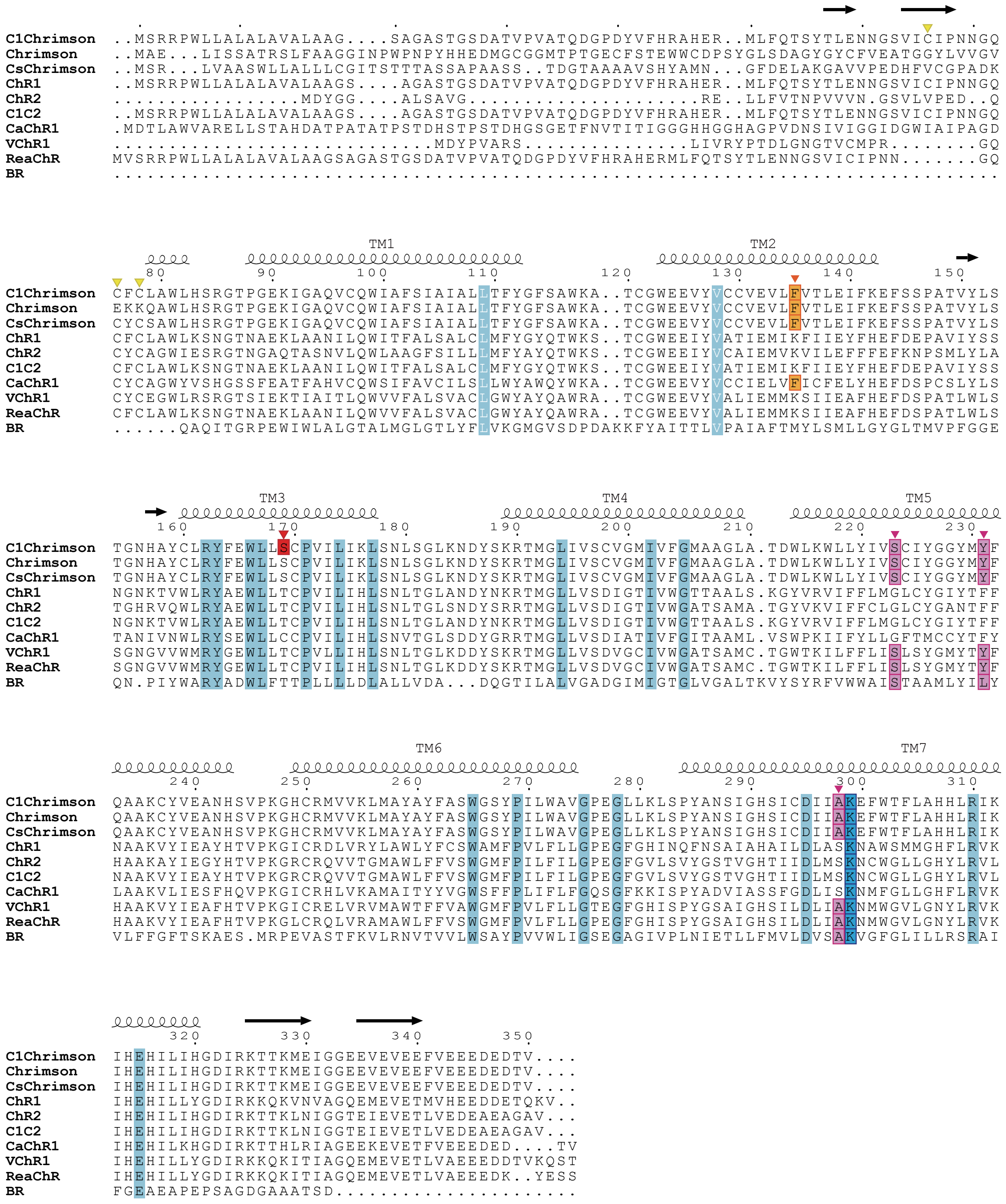

Supplementary Figure 1│Sequence alignment of C1Chrimson and ChR variants.

Amino acid sequences of C1Chrimson, Chrimson (*Cn*ChR1), CsChrimson, *Cr*ChR1, *Cr*ChR2, C1C2, *Ca*ChR1, VChR1, ReaChR, and BR are aligned. Secondary structures of C1Chrimson are indicated above the sequences. The N-terminal disulfide bond-forming cysteine residues are indicated by yellow arrowheads. The completely conserved sequences are highlighted in cyan, and the conserved lysine residues forming a linkage to the chromophore retinal are indicated in the blue panels. Residues important for the red-shift absorption are indicated by arrow heads, according to their contribution manners: counterion protonation (orange) and polarity of the retinal binding pocket (pink). The position of the red-shift-causing point mutation (S169A) is indicated (red).

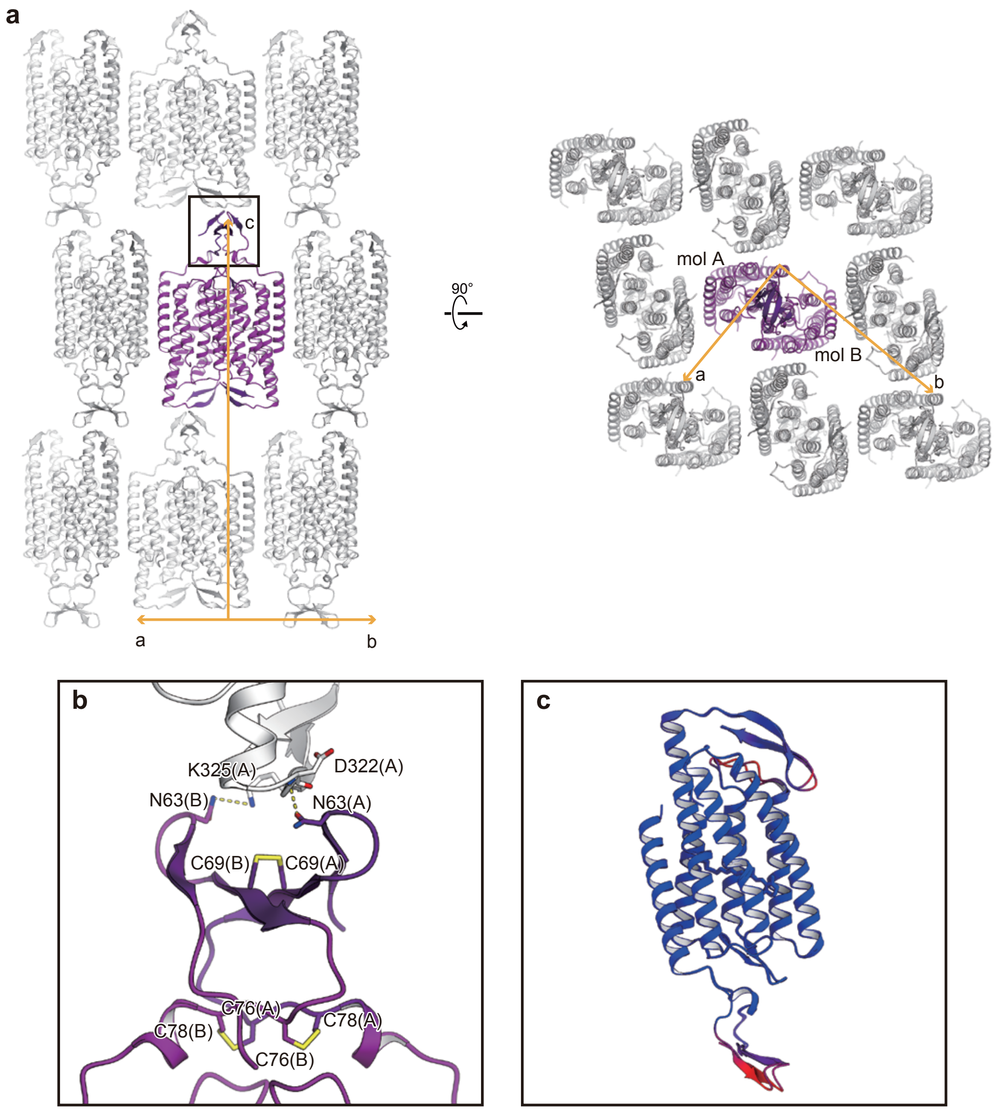

Supplementary Figure 2│Crystal packing of C1Chrimson.

a, The Chrimson proteins in the crystals are shown in ribbons, viewed from within the LCP crystal layer (left) and from the top (right). The *a*-, *b*- and *c*-axes are indicated by yellow arrows, and the two protomers (mol A and mol B) are shown. **b**, The N-terminal *Cr*ChR1-derived residues are involved in the crystal packing interactions between the layers. Disulfide bond-forming cysteine and hydrogen bonding residues are shown as sticks, with the hydrogen bonding interactions indicated by yellow dotted lines. The letters in brackets indicate the respective molecules (A and B for mol A and mol B, respectively). **c**, Two protomers are superimposed, and colored according to the distance of the C_{α} atoms between the protomers.

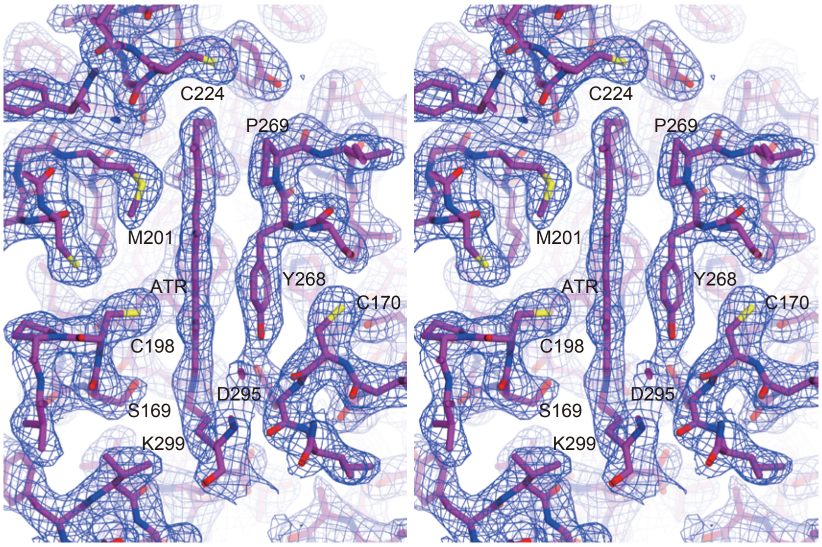

Supplementary Figure 3│Electron density of retinal.

A stereo view of the 2F_o-F_c electron density map for the retinal binding pocket is shown as a mesh representation, contoured at 1.1 σ. The all-trans retinal (ATR) and the surrounding residues are indicated by sticks.

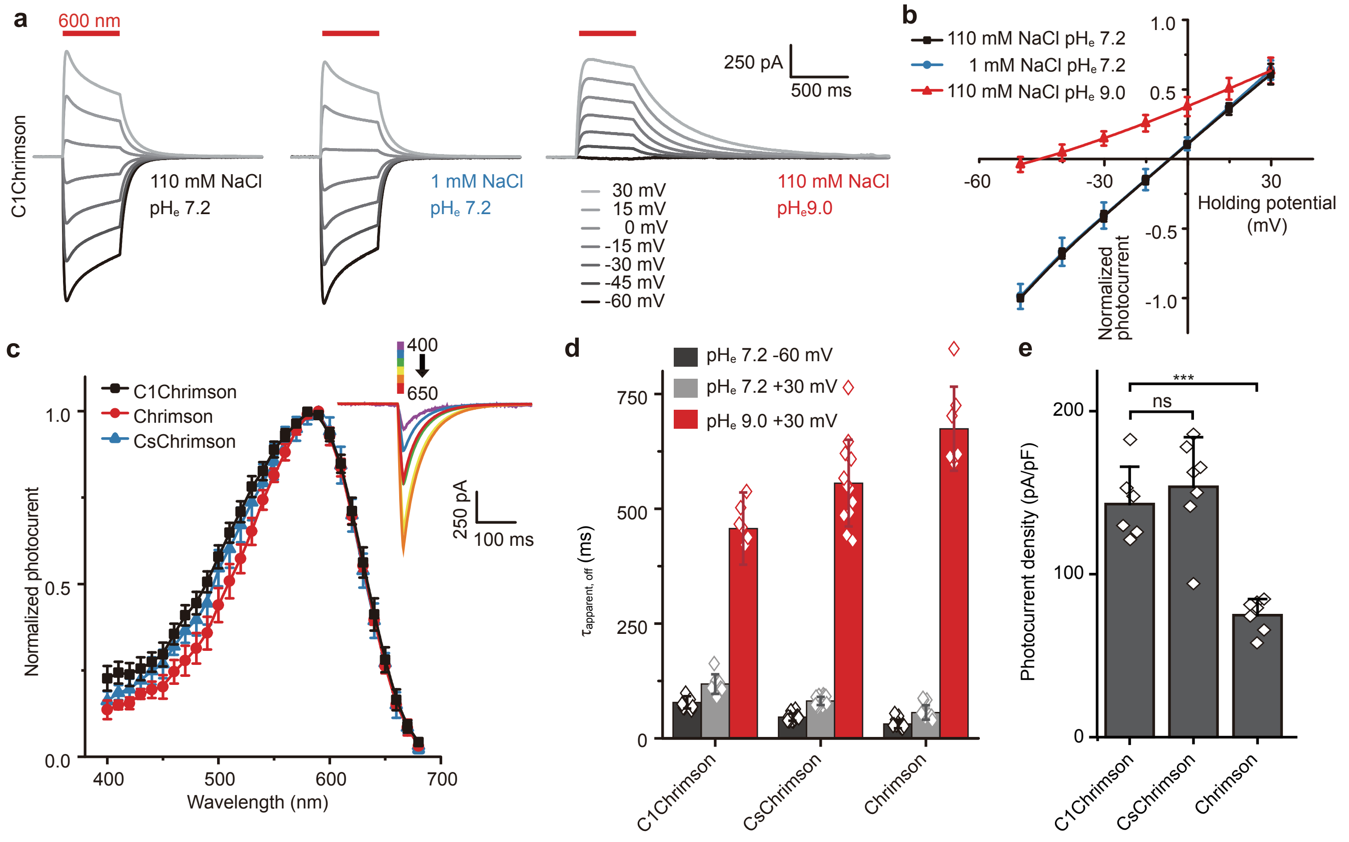

Supplementary Figure 4│Photocurrents of Chrimson variants.

a, Representative photocurrents of C1Chrimson at different voltages with different extracellular solutions of 110 mM extracellular NaCl and pH_e 7.2 (left), 1 mM NaCl and pH_e 7.2 (middle) and 110 mM NaCl and pHe 9.0 (right) with intracellular 110 mM NaCl pH_i 7.2. **b**, The current-voltage dependence of the normalized peak photocurrents of Chrimson under the ionic conditions described in (a) (Mean ± SD, n=5-16). **c**, Normalized peak photocurrents after 10 ms excitation at different wavelengths with equal photon count (Mean ± SD; n=6-10; Inlet: Representative photocurrents). **d**, Apparent off-kinetics ($\tau_{apparent,\ off}$) at positive and negative voltages and pH_e 7.2 and pH_e 9.0 (Mean ± SD; n=7-16). (**e**) Head-to-head comparison of photocurrent amplitudes of the Chrimson variants at -60 mV in symmetric 110 mM NaCl and $pH_{e,i}$ 7.2 (Mean ± SD, n=5-7). Significance was assessed by t-tests using Welch's correction in Origin 9.1®, with significance thresholds of $p < 0.05$ (*), $p < 0.01$ (**), $p < 0.001$ (***), and $p < 0.0001$ (****). p=0.5 for C1Chrimson and CsChrimson and p=0.0003 for C1Chrimson and Chrimson.

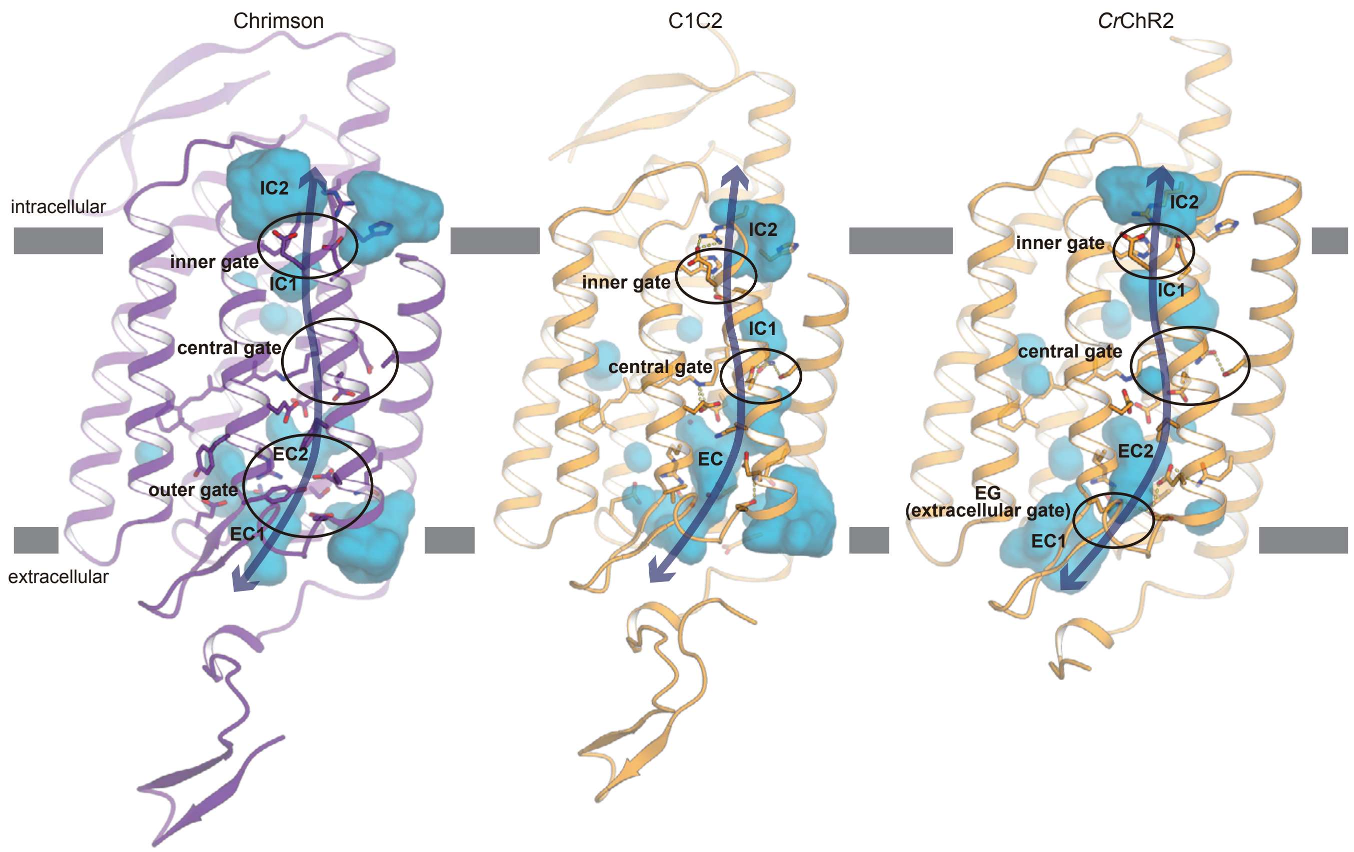

Supplementary Figure 5│Comparison of the putative ion translocating pathway.

Protein internal cavities are shown as blue surface representations, for Chrimson (left), C1C2 (center) and *Cr*ChR2 (right). By observing discontinuities and locations, we named these cavities according to the combination of extracellular cavity (EC) or intracellular cavity (IC) and the sequential numbering from the extracellular to the intracellular side. We observed three constrictions (inner, central and outer gates) and four cavities (EC1, EC2, IC1 and IC2) for Chrimson, two constrictions (inner and central gates) and three cavities (EC, IC1, and IC2) for C1C2, and three constrictions (inner, central and extracellular gates) and four cavities (EC1, EC2, IC1 and IC2) for *Cr*ChR2. Putative ion translocating pathways are indicated by navy arrows in each structure, and constriction sites are highlighted by circles.

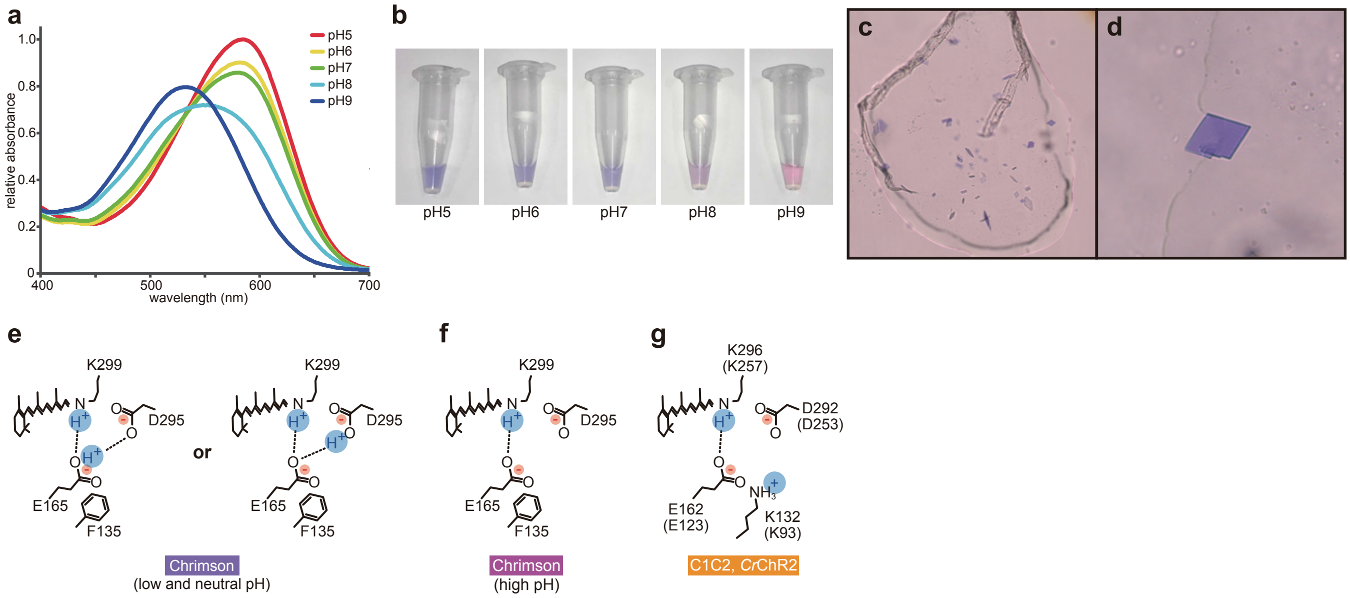

Supplementary Figure 6 │ pH-dependent peak shift and Schiff base environment.

a-**b**, Absorption spectra of the purified wild type Chrimson protein measured at the respective pH conditions (a). The color of the retinal chromophore is highly dependent on the solution pH. The Chrimson protein is blue at a lower pH, and red at higher pH. **c**-**d**, Crystals of Chrimson were obtained under low pH conditions, and accordingly the color of the crystal was blue. **e**-**g**, Proposed hydrogen bonding networks around the protonated Schiff base for Chrimson at low to neutral pH (e) and at high pH (f) and in C1C2 and *Cr*ChR2 (g).

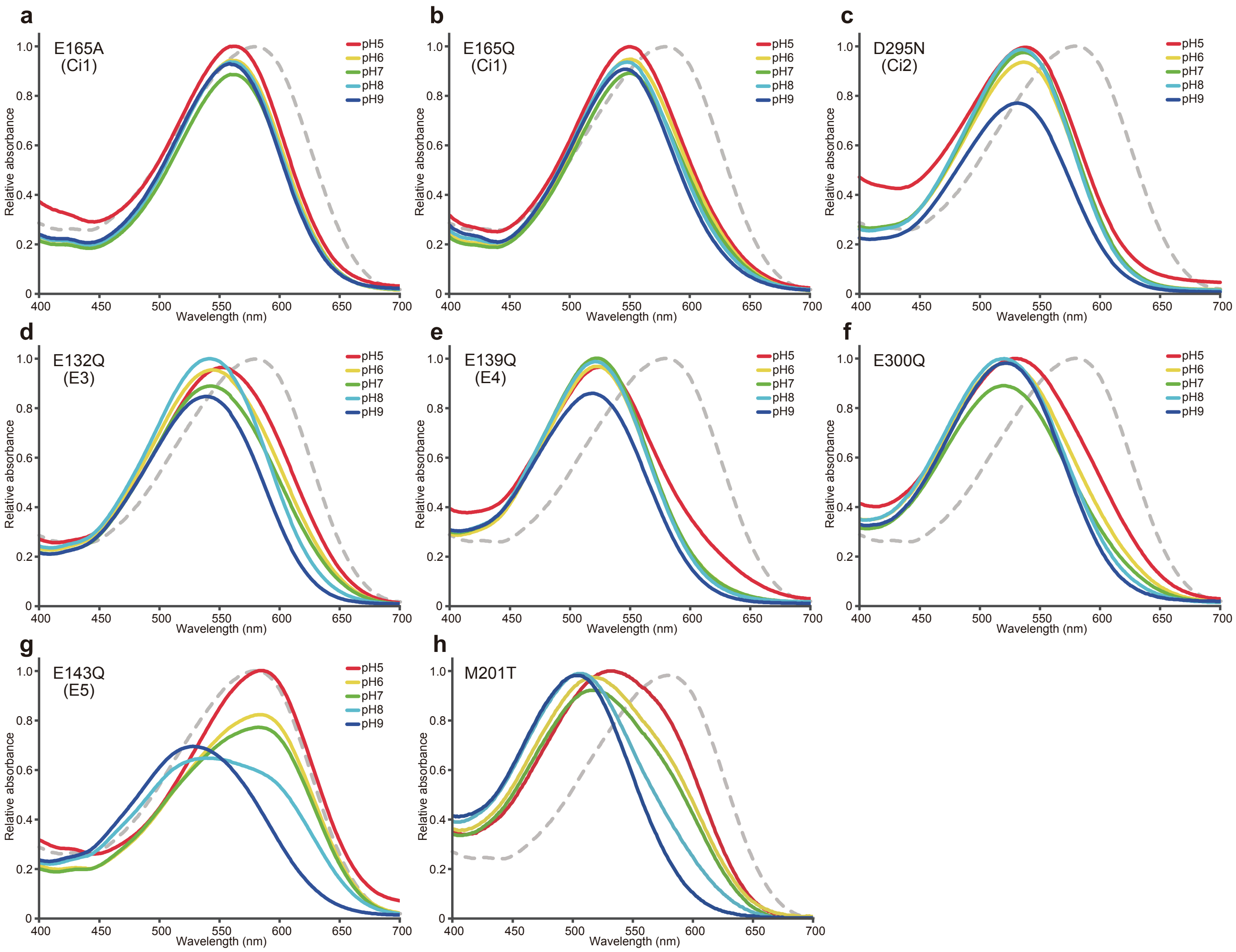

Supplementary Figure 7 | Absorption spectra of Chrimson mutants.

a-**h**, Absorption spectra of Chrimson mutants measured at different pH conditions are shown. Gray dotted lines indicate the absorption spectrum of wild type Chrimson at pH 7. The mutations of E165, E132, E139 and E300 all caused large blue-shifted absorption, while the mutation of E143 did not affect either the absorption spectrum or pH-dependency. The M201T mutant showed the largest blue-shift among the tested mutants.

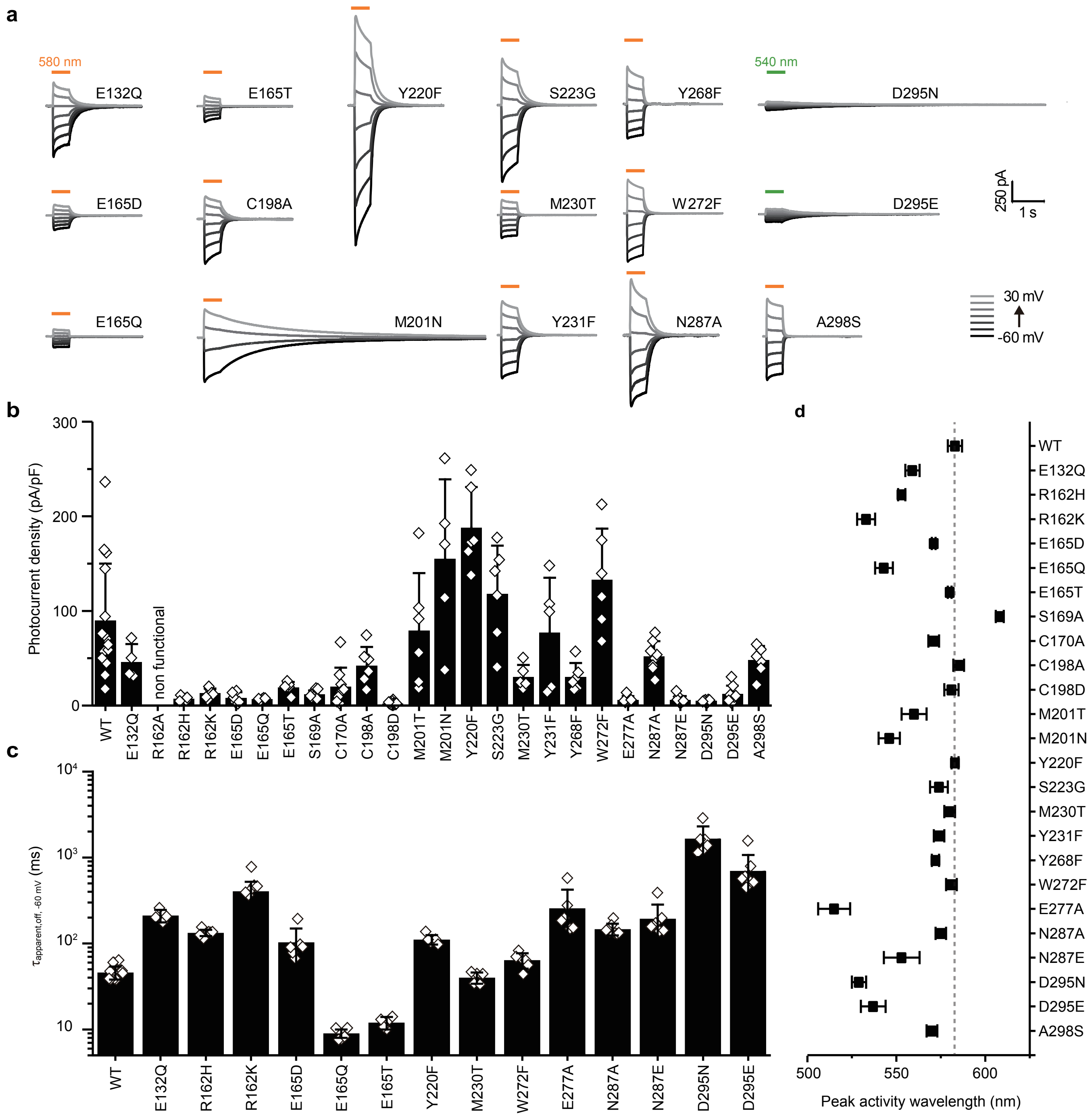

Supplementary Figure 8 | Photocurrent traces and channel kinetics of mutant Chrimson.

a, Representative photocurrent traces of Chrimson mutants in symmetric 110 mM NaCl $pH_{i,e}$ 7.2 at different voltages, illuminated with either 530 nm light (green line) or 580 nm light (orange line), depending on the peak absorption. **b–d**, Characterization of Chrimson mutants. Photocurrent density (b), channel closing kinetics at -60 mV (c) and peak activity wavelength (Mean ± SD) (d) are shown.

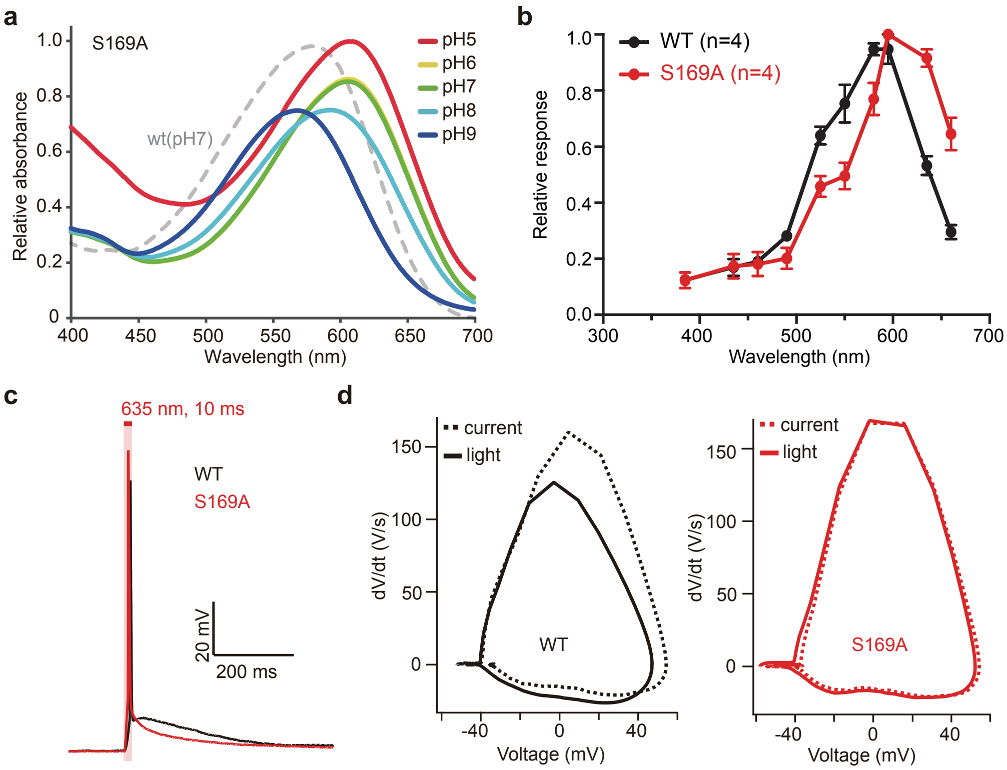

Supplementary Figure 9│pH-dependency and neuronal characterization of ChrimsonSA.

a, Absorption spectra of ChrimsonSA, measured under different pH conditions. Gray dotted lines indicate the absorption spectrum of wild type Chrimson at pH 7. **b**, Peak-normalized photocurrents in neurons expressing either WT Chrimson (black) or Chrimson-S169A (red, Mean ± SEM). **c**, Single action potentials in neurons expressing either WT Chrimson (black) or ChrimsonSA (red). **d**, Phase plots of action potentials evoked with 300 pA current injections (dashed lines) or with 8-ms red light pulses (solid lines) in neurons expressing either WT Chrimson (black) or ChrimsonSA.

Supplementary Table 1 | Data collection and refinement statistics

	Chrimson
**Data collection**	BL32XU
Space group	$P2_12_12_1$
Number of crystals	13
Cell dimensions	
a, *b*, *c* (Å)	60.99,81.44, 170.14
α, β, γ (°)	90.0, 90.0, 90.0
Wavelength	1.0000
Resolution (Å)	50-2.60 (2.69-2.60)
R_{meas}	0.144 (0.591)
$CC_{1/2}$	0.980 (0.595)
I / σI	13.8 (1.7)
Completeness (%)	100.0 (100.0)
Redundancy	9.3 (9.2)
**Refinement**	
Resolution (Å)	50-2.60 (2.69-2.60)
No. reflections	26744 (2618)
R_{work} / R_{free}	0.229/0.277 (0.313/0.358)
No. atoms	
Protein	4410
Water/Ion/Lipid	411
***B*-factors**	
Protein	85.6
Water/Ion/Lipid	80.8
**R.m.s. deviations**	
Bond lengths (Å)	0.005
Bond angles (°)	0.96
**Ramachandran plot**	
Favored (%)	95.6
Allowed (%)	4.4
Disallowed (%)	0

*Values in parentheses are for highest-resolution shell.
