# Supplementary material for: Crystal structure of the red light-activated channelrhodopsin Chrimson
Source: Nat Commun. 2018 Sep 26;9:3949. doi: 10.1038/s41467-018-06421-9 (PMC6158191; doi:10.1038/s41467-018-06421-9)
Supplement: Supplementary file 3 — Description of Additional Supplementary Files [file 41467_2018_6421_MOESM3_ESM.pdf]

Description of Additional Supplementary Files

File Name: Supplementary Data 1
Description: Primer List.
