# Supplementary material for: Crystal structure of the red light-activated channelrhodopsin Chrimson
Source: Nat Commun. 2018 Sep 26;9:3949. doi: 10.1038/s41467-018-06421-9 (PMC6158191; doi:10.1038/s41467-018-06421-9)
Supplement: Supplementary file 4 — Supplementary Data 1 [file 41467_2018_6421_MOESM4_ESM.docx]

**Supplementary Data file | primer list**

| **C1Chrimson construction** | |
| --- | --- |
| C1C2-Nt_fw | GCGCGGATCGCCATGTCGCGGAGGCCATGGC |
| C1C2-Nt_rv | GCTGTGCAGCCAAGCCAAGCAGAAGCACTGGCCG |
| CsChrimson_fw | GCTTGGCTGCACAGCAGAGG |
| CsChrimson_rv | CATGGCGATCCGCGCCCG |
|  |  |
| **Site-directed mutagenesis for absorption spectrum measurements** | |
| C1Chrimson_E165Q_fw | GCCTGCGCTACTTCCAATGGCTGCTGTCTTG |
| C1Chrimson_E165Q_rv | CAAGACAGCAGCCATTGGAAGTAGCGCAGGC |
| C1Chrimson_E165A_fw | GCCTGCGCTACTGACAATGGCTGCTGTCTTG |
| C1Chrimson_E165A_rv | CAAGACAGCAGCCATTGTCAGTAGCGCAGGC |
| C1Chrimson_D295N_fw | GGCCACAGCATCTGCAACATCATCGCCAAGGAG |
| C1Chrimson_D295N_rv | CTCCTTGGCGATGATGTTGCAGATGCTGTGGCC |
| C1Chrimson_E132Q_fw | CTACGTCTGTTGCGTCCAAGTGCTGTTCGTGAC |
| C1Chrimson_E132Q_rv | GTCACGAACAGCACTTGGACGCAACAGACGTAG |
| C1Chrimson_E139Q_fw | CTGTTCGTGACCCTGCAAATCTTCAAGGAGTTC |
| C1Chrimson_E139Q_rv | GAACTCCTTGAAGATTTGCAGGGTCACGAACAG |
| C1Chrimson_E143Q_fw | CCTGGAGATCTTCAAGCAATTCAGCAGCCCCGCC |
| C1Chrimson_E143Q_rv | GGCGGGGCTGCTGAATTGCTTGAAGATCTCCAGG |
| C1Chrimson_E300Q_fw | GACATCATCGCCAAGCAATTTTGGACCTTCCTG |
| C1Chrimson_E300Q_rv | CAGGAAGGTCCAAAATTGCTTGGCGATGATGTC |
| C1Chrimson S223G_fw | GCTGCTGTATATCGTGGGTTGCATCTACGGCGGC |
| C1Chrimson S223G_rv | GCCGCCGTAGATGCAACCCACGATATACAGCAGC |
| C1Chrimson Y220F_fw | GGCTCAAGTGGCTGCTGTTTATCGTGTCTTGCATC |
| C1Chrimson Y220F_rv | GATGCAAGACACGATAAACAGCAGCCACTTGAGCC |
| C1Chrimson A298S_fw | CTGCGACATCATCAGCAAGGAGTTTTGGACCTTCC |
| C1Chrimson A298S_rv | GGAAGGTCCAAAACTCCTTGCTGATGATGTCGCAG |
| C1Chrimson M201T_fw | CGTGTCTTGCGTGGGAACTATCGTGTTCGGCATGG |
| C1Chrimson M201T_rv | CCATGCCGAACACGATAGTTCCCACGCAAGACACG |
| C1Chrimson S169A_fw | CGAGTGGCTGCTGGCATGCCCCGTGATCCTGATC |
| C1Chrimson S169A_rv | GATCAGGATCACGGGGCATGCCAGCAGCCACTCG |
| C1C2 G220S_fw | CATTTTCTTCCTGATGTCTCTGTGCTACGGCATC |
| C1C2 G220S_rv | GATGCCGTAGCACAGAGACATCAGGAAGAAAATG |
| C1C2 F217Y_fw | CGTCCGTGTCATTTTCTATCTGATGGGCCTGTG |
| C1C2 F217Y_rv | CACAGGCCCATCAGATAGAAAATGACACGGACG |
| C1C2 S295A_fw | CCATCATTGACCTGATGGCAAAGAACTGCTGGGG |
| C1C2 S295A_rv | CCCCAGCAGTTCTTTGCCATCAGGTCAATGATGG |
| C1C2 T198M_fw | GTCAGATATCGGCATGATCGTGTGGGGCACC |
| C1C2 T198M_rv | GGTGCCCCACACGATCATGCCGATATCTGAC |
|  |  |
| **Chrimson into pmCerulean-C1 vector** | |
| Chrimson_Nhe1_for | GGATCCGCTAGCATGGCTGAGCTGATCAGC |
| Chrimson_AgeI_rev | GCATCACCGGTGCCGCCACTGTGTCCTCG |
|  |  |
| **CsChrimson into pmCerulean-C1 vector** | |
| CsChR87_Nhe1_for | GATCCGCTAGCGCCACCATGAGCAGACTGGTC |
| Chrimson_AgeI_rev | GCATCACCGGTGCCGCCACTGTGTCCTCG |
|  |  |
| **Chrimson into pAAV backbone vector** |  |
| CMV_Nhe_for | TAGTGAACCGTCAGATCCGCTAGC |
| mCerulean_HindIII_rev | GAAAAAGCTTCTACTTGTACAGCTCGTCCATGCCG |
|  |  |
| **Site-directed mutagenesis for action spectrum measurements** | |
| Chrimson_E132Q_for | CGTCTGTTGCGTCCAGGTGCTGTTCGTG |
| Chrimson_E132Q_rev | CACGAACAGCACCTGGACGCAACAGACG |
| Chrimson_R162A_for | GCCTATTGCCTGGCCTACTTCGAGTGG |
| Chrimson_R162A_rev | CCACTCGAAGTAGGCCAGGCAATAGGC |
| Chrimson_R162H_for | GCCTATTGCCTGCACTACTTCGAGTGG |
| Chrimson_R162H_rev | CCACTCGAAGTAGTGCAGGCAATAGGC |
| Chrimson_R162K_for | GCCTATTGCCTGAAATACTTCGAGTGG |
| Chrimson_R162K_rev | CCACTCGAAGTATTTCAGGCAATAGGC |
| Chrimson_E165D_for | GCCTGCGCTACTTCGACTGGCTGCTGTCTTG |
| Chrimson_E165D_rev | CAAGACAGCAGCCAGTCGAAGTAGCGCAGGC |
| Chrimson_E165Q_for | CCTGCGCTACTTCCAGTGGCTGCTGTCTTG |
| Chrimson_E165Q_rev | CAAGACAGCAGCCACTGGAAGTAGCGCAGG |
| Chrimson_E165T_for | CCTGCGCTACTTCACGTGGCTGCTGTCTTG |
| Chrimson_E165T_rev | CAAGACAGCAGCCACGTGAAGTAGCGCAGG |
| Chrimson_S169A_for | GTGGCTGCTGGCTTGCCCCGTGATC |
| Chrimson_S169A_rev | GATCACGGGGCAAGCCAGCAGCCAC |
| Chrimson_C170A_for | GGCTGCTGTCTGCCCCCGTGATCCTG |
| Chrimson_C170A_rev | CAGGATCACGGGGGCAGACAGCAGCC |
| Chrimson_C198A_for | GCCTGATCGTGTCTGCCGTGGGAATGATCG |
| Chrimson_C198A_rev | CGATCATTCCCACGGCAGACACGATCAGGC |
| Chrimson_C198D_for | CTGATCGTGTCTGACGTGGGAATGATCGTG |
| Chrimson_C198D_rev | CACGATCATTCCCACGTCAGACACGATCAG |
| Chrimson_M201T_for | GTGTCTTGCGTGGGAACGATCGTGTTCGGCATG |
| Chrimson_M201T_rev | CATGCCGAACACGATCGTTCCCACGCAAGACAC |
| Chrimson_M201N_for | GTCTTGCGTGGGAAATATCGTGTTCGGC |
| Chrimson_M201N_rev | GCCGAACACGATATTTCCCACGCAAGAC |
| Chrimson_Y220F_for | GTGGCTGCTGTTTATCGTGTCTTGC |
| Chrimson_Y220F_rev | GATCACGGGGCAAGTCAGCAGCCAC |
| Chrimson_S223G_for | GCTGTATATCGTGGGTTGCATCTACGGC |
| Chrimson_S223G_rev | GCCGTAGATGCAACCCACGATATACAGC |
| Chrimson_M230T_for | CGGCGGCTACACGTACTTCCAGGC |
| Chrimson_M230T_rev | GCCTGGAAGTACGTGTAGCCGCCG |
| Chrimson_Y231F_for | GCGGCTACATGTTCTTCCAGGCCG |
| Chrimson_Y231F_rev | CGGCCTGGAAGAACATGTAGCCGC |
| Chrimson_Y268F_for | CTTGGGGCAGCTTCCCAATCCTCTG |
| Chrimson_Y268F_rev | CAGAGGATTGGGAAGCTGCCCCAAG |
| Chrimson_W272F_for | CCCAATCCTCTTCGCAGTGGGACC |
| Chrimson_W272F_rev | GGTCCCACTGCGAAGAGGATTGGG |
| Chrimson_E277A_for | CAGTGGGACCAGCTGGACTGCTGAAG |
| Chrimson_E277A_rev | CTTCAGCAGTCCAGCTGGTCCCACTG |
| Chrimson_N287A_for | GAGCCCTTACGCAGCAAGCATCGGCCAC |
| Chrimson_N287A_rev | GTGGCCGATGCTTGCTGCGTAAGGGCTC |
| Chrimson_N287E_for | GAGCCCTTACGCAGAGAGCATCGGCCAC |
| Chrimson_N287E_rev | GTGGCCGATGCTCTCTGCGTAAGGGCTC |
| Chrimson_D295N_for | CACAGCATCTGCAACATCATCGCCAAG |
| Chrimson_D295N_rev | CTTGGCGATGATGTTGCAGATGCTGTG |
| Chrimson_D295E_for | CACAGCATCTGCGAGATCATAGCCAAGGAG |
| Chrimson_D295E_rev | CTCCTTGGCTATGATCTCGCAGATGCTGTG |
| Chrimson_A298S_for | CTGCGACATCATCTCCAAGGAGTTTTGG |
| Chrimson_A298S_rev | CCAAAACTCCTTGGAGATGATGTCGCAG |
