# Supplementary material for: Crystal structure of the red light-activated channelrhodopsin Chrimson
Source: Nat Commun. 2018 Sep 26;9:3949. doi: 10.1038/s41467-018-06421-9 (PMC6158191; doi:10.1038/s41467-018-06421-9)
Supplement: Supplementary file 2 — Peer Review File [file 41467_2018_6421_MOESM2_ESM.pdf]

Editorial Note: This manuscript has been previously reviewed at another journal that is not operating a transparent peer review scheme. The manuscript was considered suitable for publication without further review at Nature Communications.
